# Comparative Effect of UV, UV/H_2_O_2_ and UV/H_2_O_2_/Fe on Terbuthylazine Degradation in Natural and Ultrapure Water

**DOI:** 10.3390/molecules27144507

**Published:** 2022-07-14

**Authors:** José Antonio Andrades, Manuel Lojo-López, Agata Egea-Corbacho, José María Quiroga

**Affiliations:** Department of Environmental Technologies, Faculty of Marine and Environmental Sciences, University of Cadiz, Puerto Real, 11510 Cadiz, Spain; antonio.andrade@uca.es (J.A.A.); manuel.lojo@uca.es (M.L.-L.); josemaria.quiroga@uca.es (J.M.Q.)

**Keywords:** advanced oxidation processes (AOPs), terbuthylazine, natural waters, UV reactors, UPLC-MS

## Abstract

Different advanced oxidation processes (AOPs) (ultraviolet radiation, hydrogen peroxide photolysis and photo-Fenton) were applied to test the degradation of terbuthylazine in three types of water: (a) ultrapure water, (b) surface water from the Gaditana area (Los Hurones reservoir, Cádiz, Spain) and (c) groundwater from the Tempul spring in Jerez de la Frontera (Cádiz, Spain). The experiments were carried out on a laboratory scale, using two different types of reactors, batch and semi-continuous. In batch reactors, the most efficient process for the experiments carried out with both ultrapure water and underground groundwater was ultraviolet radiation, whereas for surface water from the Gaditana area, the process that obtained the best results was the photolysis of hydrogen peroxide with 2.5 mg L^−1^ of H_2_O_2_. In semi-continuous reactors, the most efficient process was the photolysis of hydrogen peroxide with 2.5 mg L^−1^ of H_2_O_2_ for all the matrices studied. In both types of reactors, terbuthylazine degradation percentages higher than 90% were achieved; the main difference was in the reaction time, which varied from minutes in the batch reactor to seconds in the semi-continuous reactor. In all the applied AOPs, N-terbutyl-6-hydroxy-N′ethyl-1,3,5-triazine-2,4-diamine (TBA-212) was generated as a reaction intermediate.

## 1. Introduction

Land use in achieving sustainable development has been highlighted by the United Nations Sustainable Development Goals (SDGs) which prioritise environmental sustainability as a way to achieve other development goals, such as alleviating poverty and hunger [1]. In particular, Goal 15 “Life on Land” has been dedicated to the protection, restoration and sustainable use of terrestrial ecosystems [2]. The increased use of herbicides brings social benefits, especially in rural areas, which see increased production and economic gains [3,4].

Despite their advantages, these synthetic compounds are, in most cases, persistent pollutants that resist biological, photochemical, chemical and biochemical degradation to varying degrees, so their half-lives in soils, surface and groundwater, aquifers, animals and even humans can be high [5]. In addition, some are considered toxic, mutagenic, carcinogenic and tumourigenic and can even cause death, thus endangering the health of the environment and society [5,6,7,8]. For all these reasons, knowledge of the levels of herbicides in surface and groundwater, as well as the limitation of their levels, has become a matter of social interest and has been taken up by European and Spanish state legislation.

Triazines are widely used herbicides in agriculture [9]. Among this group, terbuthylazine is the most persistent compound in surface environments [10] and in groundwater, its half-life varies between 263 and 366 days [11]. For this reason, Directive 2000/60/EC of the European Parliament and the Council, of 23 October 2000, established a framework for Community action in the field of water policy and its Spanish transposition, Royal Decree 817/2015, of 11 September, which establishes the criteria for the monitoring and assessment of the surface water status and environmental quality standards, as in ANNEX V [12,13]. For environmental quality standards for preferential substances, terbuthylazine is one of the parameters to be monitored in inland surface waters and other inland waters, with a maximum value of 1 µg/L as an annual average. Along these lines, European food safety authorities have reported that terbuthylazine poses a high risk to mammals and aquatic animals [14,15] and may have genotoxic effects [16]. Despite this, in recent years, its use in the European market has been re-evaluated and approved until 2021; however, in the Commission Implementing Regulation (EU) 2019/291 of 19 February 2019, its use was extended until 31 December 2024 [17].

In view of the above and the fact that the usual water treatment methods are sometimes insufficient to reduce the content of these contaminants in the water supply, it is of great interest to investigate other treatment technologies that achieve high reductions of this compound [18]. Advanced Oxidation Processes (AOPs) may constitute a suitable alternative for the removal of terbuthylazine in different matrices. AOPs are processes that involve the generation and use of trans-attractive species, mainly the hydroxyl radical (HO^●^), which is a powerful oxidising agent. This radical has suitable properties to attack all organic compounds and reacts between 10^6^ to 10^12^ times faster than alternative oxidants, such as ozone, and even faster than hypochlorite, hydrogen peroxide or chlorine, transforming them, if oxidation is complete, into CO_2_ and water [19]. Several authors also consider the half-lives of HO^●^ radicals compared to hypochlorite, hydrogen peroxide or chlorine [20,21]. Furthermore, Álvarez et al. [22] studied several treatments that degrade terbuthylazine through different photochemical processes that require an artificial light source, such as low-pressure ultraviolet lamps. However, most of these methods require long periods of time and rarely achieve the degradation of the herbicides [23,24].

The UV radiation and H_2_O_2_ process is an advanced oxidation process that generates hydroxyl radicals. This technology has been applied successfully to eliminate other pesticides and emerging contaminants [25,26]. On the other hand, Sanlaville et al. [27] in their studies showed that, in the presence of H_2_O_2_ and UV radiation, terbuthylazine in its degradation process allows the formation of amelin.

Although there are some documents on the subject, there is still a paucity of information about the behaviour of different technologies in different arrays and the optimisation of performance for each array. In the present work, the efficiency of three advanced oxidation processes: (i) UV irradiation, (ii) UV/H_2_O_2_ and (iii) UV/H_2_O_2_/Fe for the degradation of terbuthylazine was evaluated. A comprehensive study of the effect of the water matrix and the design of the reactor used on the efficiency of the various technologies applied was carried out. The oxidation processes were applied to terbuthylazine in three different water matrices and in two types of reactors (a Batch reactor (LUZCHEM) and a semi-continuous reactor). The evolution of TOC and chloride ions during the processes studied was also monitored and the reaction intermediates of terbuthylazine after the application of AOPs in ultrapure water were determined.

## 2. Materials and Methods

### 2.1. Photoreactors

The terbuthylazine degradation processes were carried out in two different types of reactors: batch and semi-continuous. Each of them is described in detail below.

**Figure 1:**
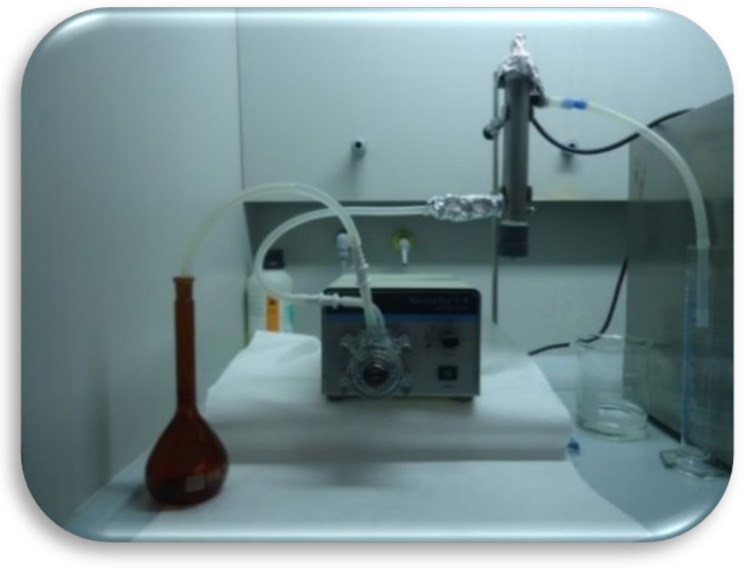
Semi-continuous reactor used for the degradation of terbuthylazine in ultrapure water, groundwater and water from the Gaditana area.

(i) Batch-type ultraviolet reactor: Laboratory-scale complete mix photoreactor from the commercial company LUZCHEM, equipped with 14 low-pressure mercury lamps (254 nm), with a lamp intensity of 0.15 w m^−2^ and an average illumination of 175 foot-candles. It is equipped with a temperature control system, exposure time, irradiation power and stirring system. Before starting the experiments, the equipment was left on for 10 min to stabilise the light intensity of the lamps. The samples, previously doped with 0.5 mg L^−1^ terbuthylazine, were placed in a 50-mL quartz reactor and introduced into the photoreactor, always in the same position, controlling the irradiation time and the power of the lamps.
(ii) Semi-continuous UV reactor: This reactor consists of a vessel in which the sample to be treated is deposited and sucked in by a peristaltic pump with a flow control system that pumps the sample into the reactor where it is treated with UV radiation (Figure 1). In this case, UV-C light provided by a low-pressure mercury lamp with a power of 40 W is used. The speed control of the pump controls the flow rate, which makes it possible to work with different exposure times or different flow rates and, thus, to determine the dose received at each moment.

### 2.2. Studied Matrices

The degradation of terbuthylazine was studied in three aqueous matrices: (i) ultrapure water (Milli-Q, 18.2 MΏ cm), (ii) surface water from the Gaditana area (Hurones reservoir) and (iii) groundwater from the Tempul spring in Jerez de la Frontera (spring water). The aim was to determine the possible effects of the matrix on the behaviour of the pesticide. Table 1 shows some characteristics of the aqueous samples studied.

### 2.3. Applied Advanced Oxidation Processes

The advanced oxidation processes applied in the present work were (i) ultraviolet (UV) irradiation, (ii) photolysis of H_2_O_2_ with UV irradiation (H_2_O_2_/UV) and (iii) photo-Fenton with UV irradiation (FeSO_4_/H_2_O_2_/UV). Table 2 shows the experimental conditions under which the tests were carried out. For the hydrogen peroxide photolysis experiments, the pH of the terbuthylazine solution was first adjusted, and then 2.5 mg L^−1^ H_2_O_2_ was added. For the photo-Fenton processes, the iron source (0.25 mg L^−1^) was first added to the reactor containing terbuthylazine, then the pH was measured and the hydrogen peroxide was added. During the whole process, the samples were stirred and the temperature reached in the reactors did not exceed 28 °C in any case.

Samples were taken at pre-set time intervals within the pesticide degradation process (Table 2) and subsequently measured according to the analytical techniques specified in Section 2.3.

### 2.4. Analytical Methods

The terbuthylazine concentration during the degradation processes was monitored by High Performance Liquid Chromatography (HPLC), using the Beckman–Coulter System Gold 125 Solvent Module. The chromatograph is equipped with a System Gold 166 UV detector and a Licrospher 100 Rp-18 (5 µm) pre-column. A C18 Lichrocart 5 µm, 2 mm × 125 mm, Superspher 100 RP-18 e column was used as the stationary phase.

The mobile phase used was acetonitrile and ultrapure water in the ratio 70:30 (*v*/*v*), with a flow rate of 1 mL min^−1^. The analyses were carried out with an injection volume of 20 µL using a Hamilton ga 22S/51 mm/pst2 syringe (SUPELCO). Terbuthylazine detection was performed at a wavelength of 230 nm. The process was at controlled room temperature.

For the detection of reaction intermediates, a Waters UPLC model ACQUITY UPLC H-Class System with a SYNAPT G2 mass spectrometry detector module, with a BEH C-18 column of 50 mm length, 2.1 mm external diameter and 1.7 mm internal diameter, was used. Intermediates were determined for all AOPs applied in ultrapure water, to avoid interference from the different wastewater components, following the times described in Table 2.

For the determinations of chloride ions, the Metrohm ion chromatimer of the Compact IC series, model 881 Compact IC pro and 882 Compact IC, equipped with an electrical conductivity detector and operated with the MagIC Net chromatography software, was used.

The UV dose applied to each sample was calculated by multiplying the exposure time by the average intensity in the system. The total exposure time was estimated by multiplying the number of cycles by the exposure time of each cycle. The intensity at each point was estimated using equations for light scattering through the quartz jacket and the fluid, considering the transmittance for the quartz to be 0.94 and measuring the experimental transmittance of the fluid to be around 0.90. The intensity was estimated by integrating the intensity over the reactor section area.

For the measurement of total organic carbon, a Shimadzu model TNM-L total organic carbon analyser (TOC-L) was used. TOC concentrations were calculated as the difference between total carbon (TC) and inorganic carbon (IC). LOD for the TOC instrument was for TC and IC, 1–4.000 mg L^−1^ and 0.25–600 mg L^−1^, respectively. In all cases, the correlation coefficient was at least 0.99. The pH and conductivity of the pesticide solutions were measured with a Crison MM4 multimeter. The initial and final concentration of hydrogen peroxide was followed using a titanium oxysulphate solution, measured by a spectrophotometric method at 410 nm (modified DIN 38 402 H15 method), in a JENWAY model 7315 spectrophotometer.

The samples were previously filtered using 0.45 µm fiberglass filters so that the presence of suspended solids would not influence the transmittance of UV radiation, which, in all cases, was greater than 90%.

### 2.5. Reagents

Terbuthylazine was supplied by Supelco with a purity of ≥98.0%. Table 3 shows the main characteristics of this compound.

Iron sulphate heptahydrate for analysis (FeSO_4_-7H_2_O) with a purity of 99.5%, as well as 30% *w*/*v* hydrogen peroxide were provided by MERCK. Catalases used to stop the reaction of hydrogen peroxide, titanium oxysulphate and sodium bisulphite were supplied by MERCK. Acetonitrile and other HPLC-grade reagents and solvents were supplied by Prolabo (VWR). The 0.45 µm fiberglass filters were supplied by Pall Corporation.

### 2.6. Data Processing

The advanced oxidation processes applied to terbuthylazine in the three water matrices were fitted by means of first-order degradation kinetics, described by the following equation:

C = C_o_ · e^−kt^ (1)

where C is the concentration of terbuthylazine according to each time interval (t) and C_o_ is the initial concentration of terbuthylazine.

## 3. Results and Discussion

Prior to the introduction into both types of reactors (batch reactor and semi-continuous reactor), the three aqueous matrices (ultrapure water, surface water and groundwater) were doped with 0.5 mg/L terbuthylazine. Each water sample, in each reactor, was subjected to the three oxidation processes studied (UV radiation, hydrogen peroxide photolysis and photo-Fenton). The duration of the experiments in the batch reactor with UV radiation was 10 min, while the duration of the hydrogen peroxide photolysis and photo-Fenton processes was 5 min. This time difference is due to the fact that the photolysis and photo-Fenton processes are faster than UV radiation because a catalyst is included in the process [28,29]. For the tests in the batch reactor and in the three matrices studied, the processes were applied for 120 s (2 min). Table 4 shows the evolution of the degradation percentages and chloride ions, TOC and H_2_O_2_ consumption in the oxidation processes applied for the LUZCHEM and semi-continuous reactors for the three matrices studied.

### 3.1. Experiments Carried out in a Batch Reactor

**Figure 2:**
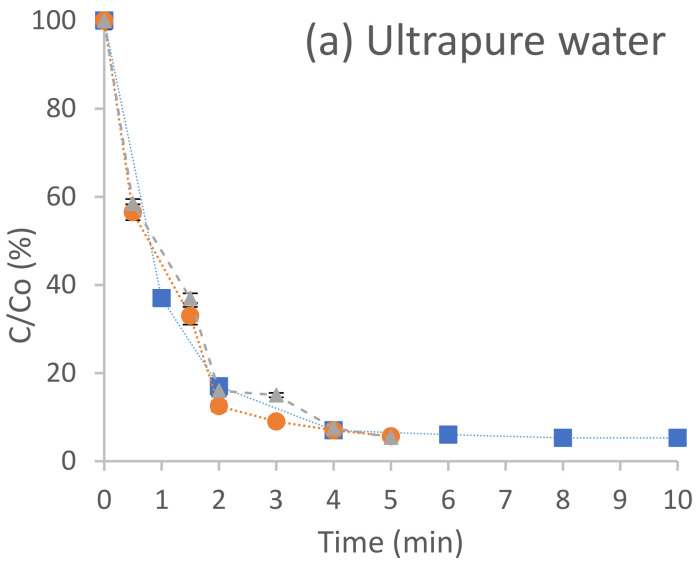

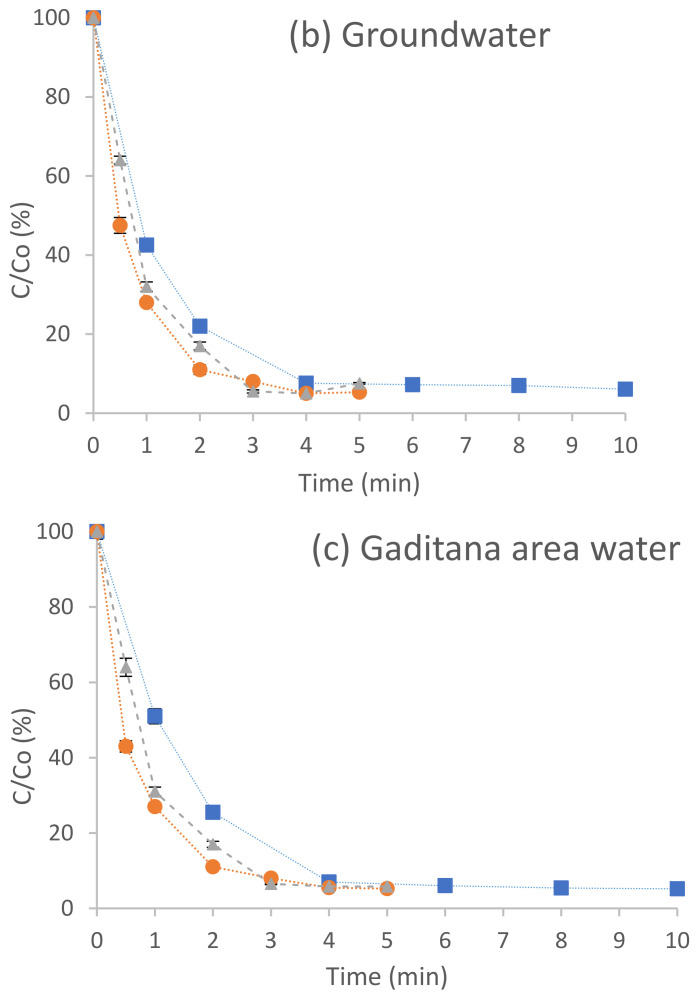
Degradation of the pesticide terbuthylazine in ultrapure water (**a**), groundwater (**b**) and water from the Gaditana area (**c**) by ultraviolet radiation (■), hydrogen peroxide photolysis (●) and photo-Fenton (▲) in a batch reactor.

Figure 2 shows the percentage degradation of terbuthylazine, considering the concentration in each time interval (C) with respect to the initial concentration Co (C/C_o_, %), as a function of time (min), after the application of ultraviolet radiation (■), hydrogen peroxide photolysis (●) and photo-Fenton (▲) treatments in ultrapure water (Figure 2a), groundwater (Figure 2b) and water from the Gaditana area (Figure 2c). Table 5 shows the values of the kinetic constants of the AOPs applied in the batch reactor for the three aqueous matrices studied.

#### 3.1.1. Degradation of Terbuthylazine in Ultrapure Water Carried out in a Batch Reactor

In ultrapure water (Figure 2a), a similar behaviour was observed for the three applied processes, reaching approximately 94% degradation after 5 min of exposure. The applied processes followed the order of degradation UV < H_2_O_2_/UV ≈ FeSO_4_/H_2_O_2_/UV (Figure 2a).

UV radiation oxidises terbuthylazine by both direct and indirect mechanisms of action, reaching a degradation rate of 0.010 min^−1^. Direct photolysis occurs when UV light attacks terbuthylazine directly [30]. This process occurs through the electronic excitation of the organic substrate, which allows an electron transfer from the excited state of the substrate to molecular oxygen or homolysis of the ground state to form organic radicals that react with oxygen. Indirect oxidation occurs when radicals originating in the aqueous medium under the action of UV radiation attack the terbuthylazine molecules. A further application of UV radiation for 5 min does not increase the degradation rate, indicating that UV radiation alone is not sufficient to further oxidise the compound [31]. Prada-Vásquez [32] reported that terbuthylazine removal was significantly accelerated by the presence of solar radiation in the ozonation process.

The applied hydrogen peroxide and photo-Fenton photolysis processes showed a similar behaviour, reaching, at 5 min, 94.34% and 94.60% degradation, respectively. For both processes, in which the degradation rate was in the order of 0.012 min^−1^, although the generation of hydroxyl radicals increased the reaction rate slightly, an increase in the degradation rate was not implied. This may be because hydroxyl radicals (HO^●^), being highly energetic and not very selective oxidants, can be captured by other species in the reaction medium that are susceptible to oxidation [33]. Among these species would be the chloride ions released as the pesticide is broken down, which would capture part of the hydroxyl radicals generated, thus reducing the degradation rate of the process. The reaction intermediates formed are also attacked by hydroxyl radicals.

For the photo-Fenton process, although a high degradation rate of terbuthylazine is achieved, it does not become more efficient because the treated water has a pH of 5.59, which reduces the Fe^+2^ available to increase the production of hydroxyl radicals. Iron precipitates in solution, reducing the degradation rate through the formation of iron hydroxy-complexes [34]. If the reaction had taken place at pH < 3, the reaction would be autocatalytic [35], as Fe(III) decomposes H_2_O_2_ into O_2_ and H_2_O via a chain mechanism, as proposed by Haber and Weiss [36], forming HO^●^ by reaction (2), which then reacts by oxidising Fe(III) or attacking organic matter:

Fe^+2^ + H_2_O_2_ → Fe^+3^ + OH^−^ + HO^●^   k = 76 L mol^−1^ s^−1^ (2)

Fe^+2^ + HO^●^ → Fe^+3^ + OH^−^ (3)

RH + HO^●^ + H_2_O → ROH + H_3_O^+^ → oxidised products (4)

TOC values show different behaviours depending on the AOP studied. Monitoring the process using this tool is important because TOC values close to zero are the only ones that guarantee that recalcitrant pollutants or intermediates of a greater persistence and toxicity than the initial ones do not accumulate. In the case of treatment with ultraviolet radiation, there is a decrease in TOC from 3.87 mg L^−1^ to 1.76 mg L^−1^ (Table 4), indicative of the generation of reaction intermediates and that the total mineralisation of the compound does not occur. For photolysis and photo-Fenton, the remaining TOC content is 75.0% and 81.1%, respectively, indicative of a high degree of compound mineralisation.

On the other hand, the evolution of chloride ions in solution (Table 4) remained unchanged throughout the oxidation processes, which may indicate that the compounds that capture OH radicals are not these ions, but the intermediate organic molecules formed.

#### 3.1.2. Degradation of Terbuthylazine in Groundwater (Spring Water) Carried out in a Batch Reactor

In the groundwater experiments, the degradation order followed for terbuthylazine was UV < FeSO_4_/H_2_O_2_/UV < H_2_O_2_/UV (Figure 2b). These results were also reflected in their kinetic constants, which showed values of 0.010 min^−1^, 0.009 min^−1^ and 0.005 min^−1^, respectively (Table 5).

The photolysis of hydrogen peroxide and photo-Fenton are nearly equally efficient, reaching degradation percentages of 95% after 4 min, while ultraviolet radiation reaches 92% degradation in the same time. This behaviour may be due to the fact that the bicarbonates present in these waters, around 228 mg L L^−1^ (Table 1), can scavenge hydroxyl ions, decreasing the ions available to degrade the pesticide studied, according to the following reaction:

HO^●^ + HCO_3_^−^ → H_2_O + CO_3_^−●^ k = 8.5 × 10^6^ M^−1^ s^−1^ (5)

The carbonate radicals formed according to reaction (5) are highly selective and react relatively slowly with organic compounds [37]. These radicals can additionally react with hydrogen peroxide to form H_2_O^●^ radicals, which are much less reactive than hydroxyl radicals.

In addition to bicarbonates, natural organic matter present in natural waters acts not only as a scavenger of the hydroxyl radical, but also as a filter for incident ultraviolet light, reducing the fraction of radiation available to react with hydrogen peroxide [38,39,40], which is the major source of hydroxyl radicals in the UV/H_2_O_2_ system, leading to a reduction in the generation of these radicals.

#### 3.1.3. Degradation of Terbuthylazine in Surface Water (Gaditana Area) Carried out in a Batch Reactor

For the water oxidation tests in the Gaditana area (Figure 2c), the order followed by the degradation processes is FeSO_4_/H_2_O_2_/UV ≈ H_2_O_2_/UV > UV, giving kinetic constants of 0.011 min^−1^, 0.011 min^−1^ and 0.009 min^−1^, respectively (Table 5). The photo-Fenton process and hydrogen peroxide photolysis reach a degradation percentage higher than 90% after 3 min, not exceeding 95% after 5 min of testing, whereas with UV radiation alone, a similar degradation percentage was reached, although in a slower manner.

For this type of water, and in the photo-Fenton oxidation experiment, a decrease in TOC from 1.93 mg L^−1^ to 1.87 mg L^−1^ was observed (Table 4), which indicates the formation of reaction intermediates which capture the hydroxyl radicals, thus preventing the complete mineralisation of the pesticide.

Hydrogen peroxide photolysis is one of the most efficient advanced oxidation processes because it combines the immediate effects of ultraviolet radiation and the generation of hydroxyl radicals produced by the homolytic cleavage of H_2_O_2_, as observed in the following reactions [30,41]:

H_2_O_2_ → 2HO^●^ (6)

H_2_O_2_ + HO^●^ → HO_2_^●^ + H_2_O (7)

H_2_O_2_ + 2HO^●^ → HO^●^ + O_2_ + H_2_O (8)

2HO^●^ → HO_2_^●^ + H_2_O_2_ (9)

2HO_2_^●^ → H_2_O_2_ + O_2_ (10)

HO^●^ + HO_2_^●^ → O_2_ + H_2_O_2_ (11)

For the case of terbuthylazine oxidation in surface water by hydrogen peroxide photolysis, although there is a reduction of the initial TOC from 2.05 mg L L^−1^ to 1.85 mg L^−1^ (Table 4), it is indicative of the onset of pesticide mineralisation by a hydroxylation and dehalogenation of terbuthylazine or by a dehydration and subsequent dehalogenation of the compound [42]. The failure to exceed 95% degradation can be explained by an excess of peroxide, which continues to react with the hydroxyl radicals generated by reaction (6), generating HO_2_^●^ radicals, as observed in reaction (7). Although both HO^●^ and HO_2_^●^ radicals react indiscriminately with organic matter, the latter is less reactive, explaining the reduction in the mineralisation process of the pesticide.

The ions present in natural waters (chlorides, alkalinity, etc.) also interfere with the action of hydroxyl radicals, reducing their effectiveness and their radius of action. Alkalinity plays an important role as it sequesters hydroxyl radicals, slowing down the reaction process [43]. The bicarbonate content in this case is 167 mg L^−1^; although slightly lower than that found in groundwater (228 mg L^−1^), it also interferes with the degradation efficiency of terbuthylazine. A slight increase in available chloride was also found in the water from 43.65 mg L^−1^ to 43.98 mg L^−1^ (Table 4), which captures the hydroxyl radicals generated and prevents the process from continuing [31,44].

### 3.2. Experiments Performed in a Semi-Continuous Reactor

**Figure 3:**
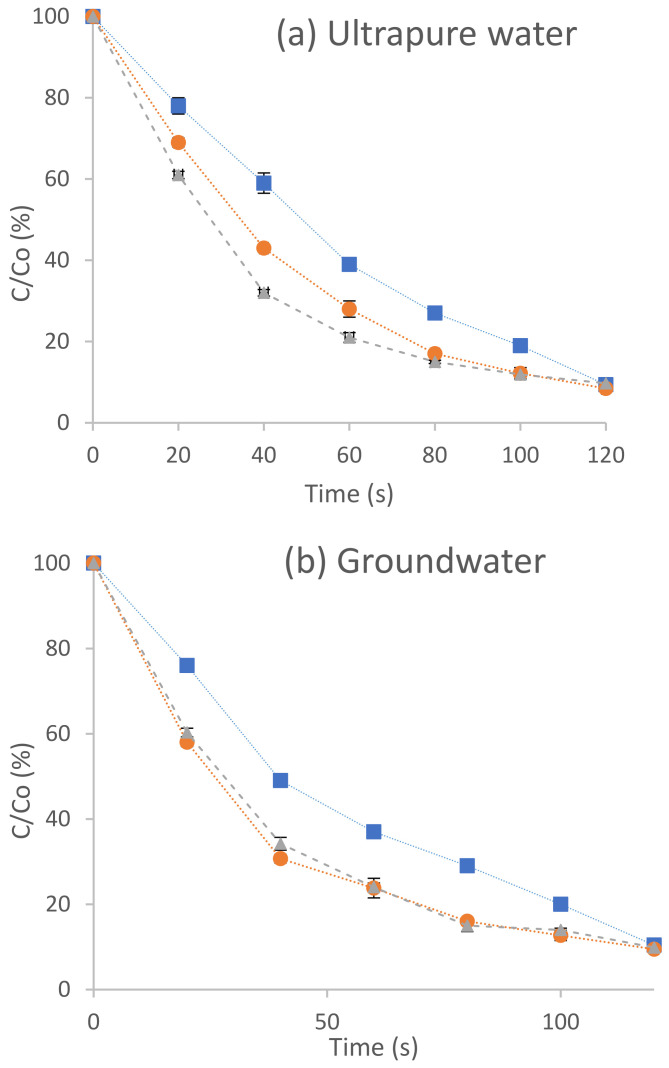

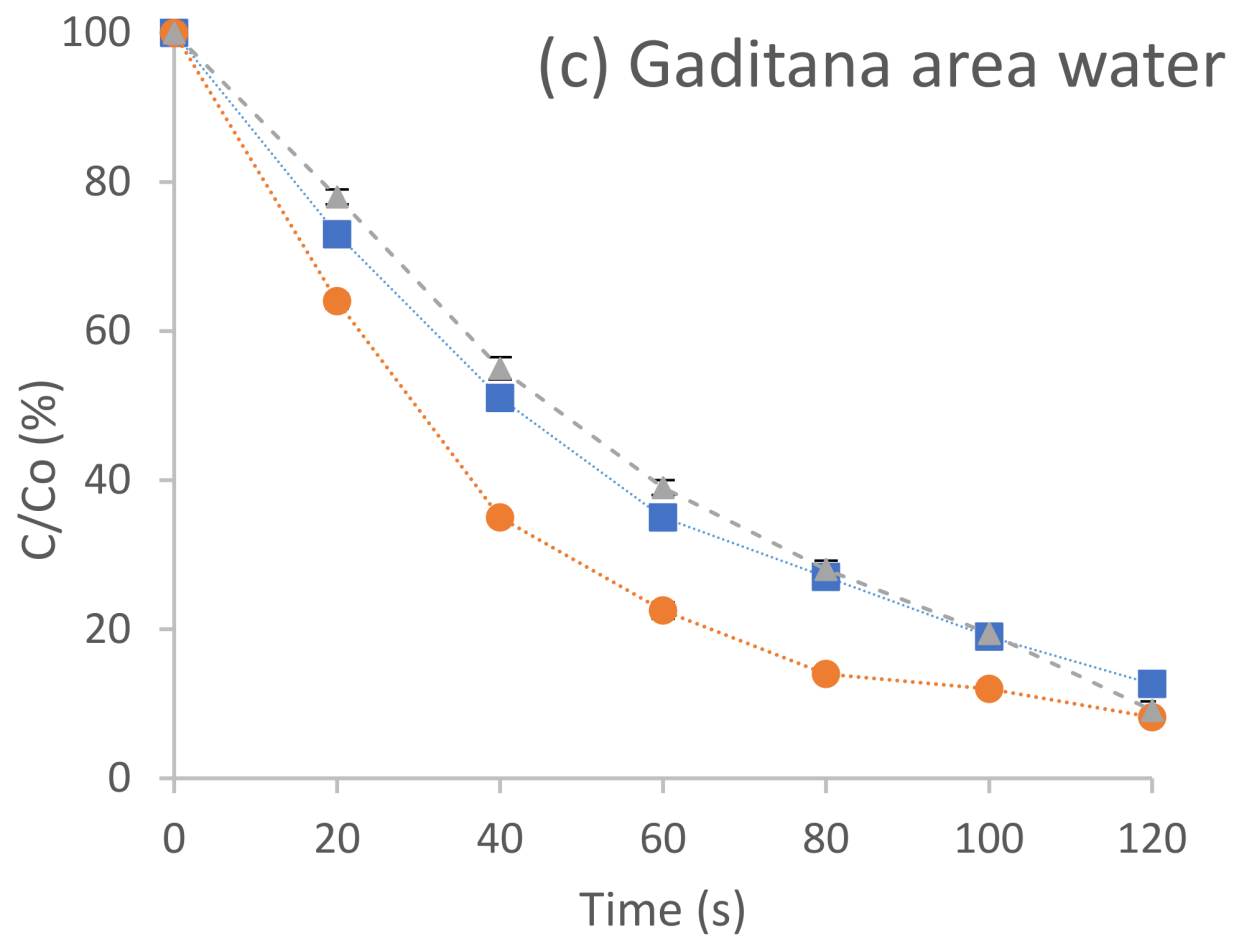
Degradation of the pesticide terbuthylazine in ultrapure water (**a**), groundwater (**b**) and water from the Gaditana area (**c**) by ultraviolet radiation (■), hydrogen peroxide photolysis (●) and Photo-Fenton (▲) in a semi-continuous reactor.

Figure 3 shows the results obtained for the degradation of terbuthylazine (C/C_o_ [%]) as a function of time (s) after the application of ultraviolet radiation (■), hydrogen peroxide photolysis (●) and photo-Fenton (▲) treatments in ultrapure water (a), groundwater (b) and water from the Gaditana area (c). In the three matrices studied, the processes were applied for 120 s (2 min). Table 5 shows the values of the kinetic constants of the AOPs applied in the semi-continuous reactor for the three aqueous matrices studied.

#### 3.2.1. Degradation of Terbuthylazine in Ultrapure Water Performed in a Semi-Continuous Reactor

For the terbuthylazine solutions in ultrapure water, 90% degradation was achieved after 120 s for all three processes applied (Figure 3a), although differences in their kinetics were observed. Thus, the photo-Fenton process is the fastest with a kinetic constant of 0.022 s^−1^ and 90.38% degradation. This is followed by hydrogen peroxide photolysis with a kinetic constant of 0.021 s^−1^ and a degradation rate of 91.56%. Finally, ultraviolet radiation shows a kinetic constant of 0.019 s^−1^ and 90.62% oxidation. Although the photo-Fenton process was the fastest, it presented a lower percentage of degradation, although it was very similar to the others. The cause may be due to the high UV-C doses applied and the precipitation of iron at a pH around 5, which darkened the sample and prevented the passage of UV radiation [45].

As can be seen in Table 4, TOC in the three processes evolved in a similar way. In the case of photo-Fenton, the TOC concentration decreased from 2.61 mg L^−1^ to 1.92 mg L^−1^, while in the other two processes applied, the final TOC concentration decreased from 2.28 mg L^−1^ to 0.45 mg L^−1^ for UV radiation and from 2.16 mg L^−1^ to 1.73 mg L^−1^ for hydrogen peroxide photolysis. These decreases could correspond to the mineralisation of the pesticide. A lower TOC decrease is observed in photo-fenton than in UV. According to Paterlini and Nogueira [46], this may be due to the fact that a high FeOx content plays a negative role when considering TOC removal. This is explained by the fact that higher concentrations of FeOx imply a higher TOC content and hence, lower mineralisation percentages.

#### 3.2.2. Degradation of Terbuthylazine in Groundwater (Spring Water) Performed in a Semi-Continuous Reactor

In groundwater (Figure 3b), the photo-Fenton and photolysis processes of hydrogen peroxide presented the same degradation kinetics, with a constant of 0.020 s^−1^, while ultraviolet radiation has a kinetic constant of 0.018 s^−1^. Although the percentage of pesticide reduction is high (the three processes reach approximately 90% degradation after 120 s), a complete mineralisation of the pesticide is not achieved due to the formation of intermediate compounds in the three processes applied. The reason may be due to the fact that, as in the batch reactor, the carbonates act as scavengers for the hydroxyl radicals generated in the process. This behaviour is evident in the TOC variations. The photolyses of hydrogen peroxide and photo-Fenton show the same kinetics, but a different decrease in TOC, 25.89% and 9.02%, respectively, compared with 30.14% for ultraviolet radiation. The groundwater shows similar behaviour in both reactors studied, whose kinetic order of degradation is as follows: UV < H_2_O_2_/UV = FeSO_4_/H_2_O_2_/UV.

#### 3.2.3. Degradation of Terbuthylazine in Surface Water (Gaditana Area) Performed in a Semi-Continuous Reactor

Finally, with waters from the Gaditana area (Figure 3c), hydrogen peroxide photolysis is the fastest process with a kinetic constant of 0.022 s^−1^, followed by photo-Fenton with a constant of 0.021 s^−1^ and ultraviolet radiation with a constant of 0.017 s^1^, i.e., H_2_O_2_/UV > FeSO_4_/H_2_O_2_/UV > UV. The highest degradation rates are reached at 100 s (90%) for photo-Fenton and photolysis, whereas for UV radiation, it is necessary to wait for 120 s to reach 88% degradation.

As for the evolution of TOC (Table 4), it remained unchanged in the treatment with ultraviolet radiation, decreased with the photolysis of hydrogen peroxide from 3.09 mg L^−1^ to 2.82 mg L^−1^ and from 4.04 mg L^−1^ to 3.33 mg L^−1^ in the photo-Fenton reaction.

Although the consumption of hydrogen peroxide in the latter two processes was not significant, the degradation rate was higher than 90%. This could be explained by the presence of organic matter in the matrix studied, which could favour the degradation processes. In natural waters, photosensitisation processes of dissolved chromophore organic matter, more specifically, of humic acids and other substances, occur and are responsible for participating in indirect photoreactions [47]. This main reaction pathway involves the excitation of organic matter and formation of reactive oxygen species involved in both energy and electron transfer and free-radical reactions [48].

Additionally, but to a lesser extent, the absorption of radiation by organic matter to a direct pathway involving its disintegration to smaller components can contribute to degradation [48].

### 3.3. Degradation Products of Terbuthylazine after Application of AOPs in Ultrapure Water

In order to determine the reaction intermediates generated when applying AOPs for the degradation of terbuthylazine, the results obtained in experiments carried out over time in ultrapure water were used. In this way, the interferences associated with the complexity of natural water matrices are avoided.

Figure S1a (Supplementary Information) shows the chromatogram obtained after applying ultraviolet radiation in the LUZCHEM reactor and Figure S1b (Supplementary Information) shows the chromatogram obtained in the semi-continuous reactor. For both reactors, two peaks are observed, the first at 2.53 min and the second at 2.98 min, which correspond to an internal pattern of Atrazine and terbuthylazine, respectively, at reaction time zero.

Before the first minute in the LUZCHEM reactor and after 20 s in the semi-continuous reactor, between 0.72 and 0.76 min, a peak appeared in the chromatogram corresponding to a reaction intermediate formed during the degradation of terbuthylazine. As the application time of the advanced oxidation process progressed, the area of the intermediate product formed increased, while that of terbuthylazine decreased. The same behaviour was observed after the application of hydrogen peroxide photolysis and photo-Fenton in both reactors.

After 4 min of irradiation in the LUZCHEM reactor, the terbuthylazine compound was completely removed, but the intermediate product remained in the solution until the end of the process, assuming 30%. On the other hand, in the semi-continuous reactor, the intermediate product appeared after 20 s and continued until the end of the process, but accounted for less than 20%. In this case, terbuthylazine also remained in the solution at 10% after 120 s.

**Figure 4:**
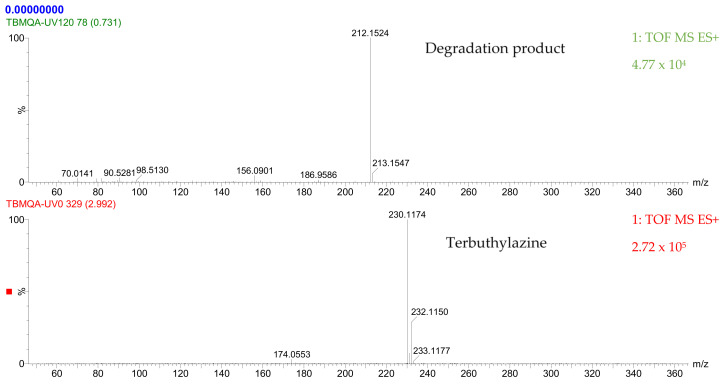
Mass chromatogram of terbuthylazine and its degradation products at time t = 0 and t = 120 s after UV irradiation in ultrapure water.

Mass spectrophotometry was used to determine the reaction intermediates detected in all the processes applied in this study. Figure 4 shows the mass chromatogram of ultraviolet radiation applied for the degradation of the pesticide in the semi-continuous reactor at the initial time, t = 0 s, and at 120 s. At the initial time, it was determined that the peak observed corresponds to terbuthylazine whose molecular weight is 230.1174 [49], while at 120 s, a product with a molecular weight of 212.1524 g/mol was determined and identified as N-terbutyl-6-hydroxy-N′ethyl-1,3,5-triazine-2,4-diamine (TBA-212).

Table 6 shows the chemical structure of the pesticide and the reaction intermediate detected, as well as its molecular mass and retention time. According to Bottoni et al. [42], the degradation of terbuthylazine can occur by an N-dealkylation of terbuthylazine, generating distilterbuthylazine (DET), and by a reduction of the chlorine atom due to a hydroxylation process, in which a substitution reaction of the halogen group by the hydroxyl group takes place, generating N-terbutyl-6-hydroxy-N′ethyl-1,3,5-triazine-2,4-diamine as in the present study.

This generated product confirms the behaviour observed in the evolution of TOC throughout the processes studied and presented in Section 3.1.1 and Section 3.2.1 and is indicative that there is no total mineralisation of the pesticide. It is necessary to remember that, for mineralisation of the pollutant to occur, not only must it disappear, but all the organic carbon must be converted into inorganic carbon in the form of CO_2_, since the TOC value is independent of the oxidation state of the compounds present in the system.

If the action of hydroxyl radicals continues, TBA-212 will form TBA-128 (amelin) and, under strong hydroxyl radical-oxidising conditions, it will be transformed into cyanuric acid [23]. These results are in agreement with those found for other triazines, such as atrazine [50].

In practice, only when the final product is a harmless compound may the partial degradation of the pollutant be acceptable. Since the product TBA-212 is considered as a compound with relevant toxicity [42], it would be necessary to continue the degradation process until total mineralisation and the formation of intermediates non-toxic to the environment.

### 3.4. Comparison of the Results Obtained in the Two Different Reactors

Although the final degradation percentages obtained for the different types of water tested for the three AOPs studied and in both types of reactors are very similar, oxidation was slower in the LUZCHEM reactor (min) than in the semi-continuum reactor (s). In the former, high percentages of degradation were obtained in 5 min, while in the latter, the same results were obtained in only 2 min (120 s). However, the contact time alone is not an appropriate parameter to compare two reactors with different geometries: it is also necessary to consider the geometry of the reactor and the transmittance of the matrices studied (90% transmittance), since it also depends on the dose of irradiation reaching the compound.

Table 7 shows the UV-C doses applied for each of the reactors. In the LUZCHEM reactor, UV-C doses between 4.5 W.s m^−2^ (0.45 mJ cm^−2^) and 90 W.s m^−2^ (9.0 mJ cm^−2^) were used, while in the semi-continuous reactor, the doses applied were between 37.4 W.s m^−2^ (3.7 mJ cm^−2^) and 224.4 W.s m^−2^ (22.44 mJ cm^−2^). Despite these differences, 100% mineralisation was not achieved in either of the two reactors or in any of the AOPs applied; a significant difference was only observed in the degree of mineralisation of the compound in the matrix studied (ultrapure water).

Thus, in the semi-continuous reactor and for ultrapure water, a TOC reduction of 80.26% was achieved compared with 54.52% in the batch reactor (LUZCHEM). This result implies that ultraviolet radiation alone is not capable of mineralising the pollutant, due to the low UV-C doses applied in the LUZCHEM reactor. For the other two matrices studied (spring water and water from the Gaditana area), the basic pH (>8 pH units) and the buffer character of this type of water mean that photolysis and photo-Fenton were not as efficient as could be expected for the doses used. Even so, degradation percentages of more than 90% were achieved in both cases.

Compared to the results obtained by other authors [51] on a groundwater matrix using a collimated beam reactor, where degradation percentages of 52% were reached with ultraviolet radiation applying a UV-C dose of 1200 mJ/cm^2^ and 95% when applying a UV-C dose of 2000 mJ cm^−2^ and 5 mg/L H_2_O_2_ (very high doses if compared to the 9 mJ cm^−2^ applied in the LUZCHEM reactor or the 22.44 mJ cm^−2^ applied in the semi-continuous reactor), in our case, the degradation rate obtained was higher than 90% in the three matrices studied.

These same authors, using the collimated reactor but with hydrogen peroxide photolysis, achieved degradation rates of 95%, slightly higher than that achieved in our study (>90%), but using a dose 100 times higher, which entails a higher energy and economic cost.

Finally, in both reactors and for all the cases studied, the same reaction intermediate compound was produced: N-terbutyl-6-hydroxy-N′ethyl-1,3,5-triazine-2,4-diamine.

## 4. Conclusions

The results presented in this paper represent an advance in the existing studies on the degradation of terbuthylazine in different matrices. In general, degradation percentages of around 90% are obtained after applying ultraviolet radiation, hydrogen peroxide photolysis and photo-Fenton in ultrapure water, surface water and groundwater. According to the results obtained, it was determined that depending on the design of the reactor, different effects of the advanced oxidation processes studied were observed. Ultraviolet radiation is the most efficient process in the LUZCHEM reactor and photolysis of hydrogen peroxide is the most efficient in the semi-continuous reactor. In addition, the matrix influences the kinetics and degradation rates of terbuthylazine. The bicarbonate content and the alkaline pH in the natural waters studied slows down the effect of the hydroxyl radical.

It was shown that the application of the AOPs studied generated the intermediate product N-terbutyl-6-hydroxy-N′ethyl-1,3,5-triazine-2,4-diamine by the direct action of ultraviolet radiation and by the hydroxyl radicals generated in the oxidation processes applied.

The study on how to correct the impact of organic compounds on UV’s influence on the elimination of terbuthylazine would open another field of study for future lines of research.

## Figures and Tables

**Table 1:** Physico-chemical characteristics of the studied matrices.

Parameter	Unit	Ultrapure Water	Surface Water	Groundwater
pH	pH units	5.57	8.43	8.35
Conductivity	µS/cm	1.3	504	560
Chloride ions	mg/L	0	24	18
Total hardness	mg/L	0	20.5	26
Bicarbonates	mg/L	0	167	228
Aluminium	mg/L	0	126	67
TOC	mg/L	0	1.3	<0.1
Terbuthylazine	µg/L	0	<0.05	<0.05

**Table 2:** Experimental conditions applied in the degradation studies of terbuthylazine in different water types.

Matrices	Process	Photoreactor	Hydrogen Peroxide (mg/L)	Fe (mg/L)	Reaction Time
Ultrapure water	Ultraviolet irradiation	LUZCHEM	--	--	0, 1, 2, 4, 6, 8, 10 (min)
		Semi-continuous UV	--	--	0, 20, 40, 60, 80, 100, 120 (s)
Surface water	Photolysis of H_2_O_2_ with UV irradiation	LUZCHEM	2.5	--	0, 0.5, 1, 2, 3, 4, 5 (min)
		Semi-continuous UV		--	0, 20, 40, 60, 80, 100, 120 (s)
Groundwater	Photo-Fenton with UV irradiation	LUZCHEM	2.5	0.25	0, 0.5, 1, 2, 3, 4, 5 (min)
		Semi-continuous UV			0, 20, 40, 60, 80, 100, 120 (s)

**Table 3:** Physico-chemical characteristics of terbuthylazine.

CAS Number	5915-41-3
Molecular formula	C_9_H_16_ClN_5_ 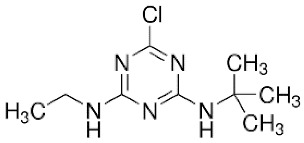
Molecular weight	229.71
Vapour pressure (mPa) at 20 °C	0.15
Density at 20 °C (g/mL)	1.1

**Table 4:** Evolution of the degradation percentages and chloride ions, TOC and H_2_O_2_ consumption in the oxidation processes applied for the LUZCHEM and semi-continuous reactors for the three matrices studied.

Photoreactor	Matrices	AOP	Degradation (%)	Initial Chloride (mg L^−1^)	Final Chloride (mg L^−1^)	Initial TOC (mg L^−1^)	Final TOC (mg L^−1^)	Initial H_2_O_2_ (mg L^−1^)	Final H_2_O_2_ (mg L^−1^)
LUZCHEM	Ultrapure water	UV	94.71	2.36	2.40	3.87	1.76	--	--
		UV + H_2_O_2_	94.34	2.36	2.40	3.44	2.58	2.53	2.24
		UV + H_2_O_2_ + Fe	94.60	2.35	2.38	2.64	2.14	2.19	0.23
	Groundwater (Tempul spring)	UV	94.83	18.90	19.09	2.18	1.73	--	--
		UV + H_2_O_2_	94.78	18.50	18.65	2.33	1.86	2.49	2.00
		UV + H_2_O_2_ + Fe	94.20	18.82	18.80	2.43	2.20	2.24	1.57
	Surface Water (Gaditana area)	UV	93.93	43.65	43.98	3.13	3.03	--	--
		UV + H_2_O_2_	94.69	42.79	42.86	2.05	1.85	2.49	1.86
		UV + H_2_O_2_ + Fe	92.45	43.23	43.28	1.93	1.87	2.10	1.43
Semi-continuous	Ultrapure water	UV	90.62	2.34	2.38	2.28	0.45	--	--
		UV + H_2_O_2_	91.56	2.44	2.48	2.16	1.73	2.57	2.24
		UV + H_2_O_2_ + Fe	90.38	2.39	2.41	2.61	1.92	2.27	1.94
	Groundwater (Tempul spring)	UV	89.59	19.03	19.47	0.73	0.51	--	--
		UV + H_2_O_2_	90.53	18.50	18.44	2.24	1.66	2.43	2.29
		UV + H_2_O_2_ + Fe	90.14	18.81	18.72	1.66	1.51	1.95	1.62
	Surface water (Gaditana area)	UV	87.34	26.13	26.02	1.65	1.65	--	--
		UV + H_2_O_2_	91.64	24.93	24.91	3.09	2.82	2.57	2.57
		UV + H_2_O_2_ + Fe	90.88	25.04	24.91	4.04	3.33	2.14	2.10

**Table 5:** Degradation kinetics of terbuthylazine after ultraviolet (UV) radiation, hydrogen peroxide photolysis (UV/H_2_O_2_) and photo-Fenton (UV/H_2_O_2_/Fe) in ultrapure water, groundwater and water from the Gaditana area in a batch reactor (k, min^−1^) and in a semi-continuous reactor (k, s^−1^).

Photoreactors	AOPs	Matrices			Kinetic Constants
		Ultrapure Water	Groundwater	Surface Water	
LUZCHEM	UV	0.010	0.005	0.009	k, min^−1^
	UV/H_2_O_2_	0.012	0.010	0.011	
	UV/H_2_O_2_/Fe	0.012	0.009	0.011	
Semi-Continuous	UV	0.019	0.018	0.017	k, s^−1^
	UV/H_2_O_2_	0.021	0.020	0.022	
	UV/H_2_O_2_/Fe	0.022	0.020	0.021	

**Table 6:** Intermediate product detected after application of AOPs to ultrapure water in the LUZCHEM and semi-continuous reactors.

Composition Elemental	Exact Mass (*m*/*z*)	Retention Time (min)	Proposed Structure and Name
TBA C_9_H_16_ClN_5_	230.1161	2.98	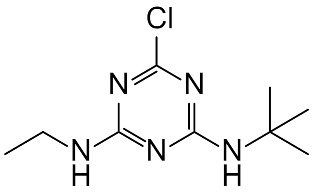 N-tert-butyl-6-chloro-N′-ethyl-1,3,5-triazine-2,4-diamine; (terbuthylazine)
2-OH-TBA C_9_H_17_N_5_O	212.1508	0.75	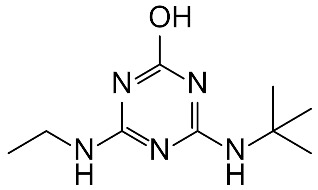 N-tert-butyl-6-hydroxy-N′-ethyl-1,3,5-triazine-2,4-diamine

**Table 7:** The UV doses applied according to the LUZCHEM reactor and semicontinuous reactor.

Photoreactors	Lamp Intensity (W m^−2^)	Time (s)	Doses UV-C (W.s m^−2^)
LUZCHEM	0.15	30	4.5
		60	9.0
		120	18.0
		180	27.0
		240	36.0
		300	45.0
		600	90.0
Semi-continuous	1.87	20	37.4
		40	74.8
		60	112.2
		80	149.6
		100	187.0
		120	224.4

## Supplementary Materials

The following supporting information can be downloaded at: https://www.mdpi.com/article/10.3390/molecules27144507/s1, Figure S1: Chromatogram of terbuthylazine degradation over time in the LUZCHEM reactor (a) and in the semi-continuous reactor (b) after application of UV radiation in ultrapure water.

## References

[1] United Nations (2015). Transforming Our World by 2030: A New Agenda for Global Action Zero. Draft of the Outcome Document for the UN Summit to Adopt the Post-2015 Development Agenda.

[2] Wolff S., Schrammeijer E.A., Schulp C.J.E., Verburg P.H. (2018). Meeting global land restoration and protection targets: What would the world look like in 2050?. Glob. Environ. Chang..

[3] Peterson M.A., Collavo A., Ovejero R., Shivrain V., Walsh M.J. (2017). The challenge of herbicide resistance around the world: A current summary. Pest. Manag. Sci. Spec. Issue Glob. Herbic. Resist. Chall..

[4] Talaviya T., Shah D., Patel N., Yagnik H., Shah M. (2020). Implementation of artificial intelligence in agriculture for optimisation of irrigation and application of pesticides and herbicides. Artif. Intell. Agric..

[5] Huang X., He J., Yan X., Hong Q., Chen K., He Q., Zhang L., Liu X., Chuang S., Li S. (2017). Microbial catabolism of chemical herbicides: Microbial resources, metabolic pathways and catabolic genes. Pestic. Biochem. Physiol..

[6] CHL (2014). Report Proposal for Harmonised Classification and Labelling Based on Regulation.

[7] Lovaković B.T., Pizent A., Kašuba V., Kopjar N., Micek V., Mendaš G., Dvoršćak M., Mikolić A., Milić M., Žunec S. (2017). Effects of sub-chronic exposure to terbuthylazine on DNA damage, oxidative stress and parent compound/metabolite levels in adult male rats. Food Chem. Toxicol..

[8] United States Environmental Protection Agency (USEPA) (2012). Registration Eligibility Decisions (REDs) Database on Tterbuthylazine (5915-41-3). (USEPA 738-R-95-005). https://archive.epa.gov/pesticides/reregistration/web/pdf/2645.pdf.

[9] Wang X., Liu Q. (2020). Spatial and temporal distribution characteristics of triazine herbicides in typical agricultural regions of Liaoning, China. Bull. Environ. Contam. Toxicol..

[10] Tasca A.L., Puccini M., Clematis D., Panizza M. (2019). Electrochemical removal of Terbuthylazine:Boron-Doped Diamond anode coupled with solid polymer electrolyte. Environ. Pollut..

[11] Navarro S., Vela N., Giménez M.J., Navarro G. (2004). Persistence of four s-triazine herbicides in river, sea and groundwater samples exposed to sunlight and darkness under laboratory conditions. Sci. Total Environ..

[12] (2000). Council Directive 2000/60/EC of the European Parliament and of the Council of 23 October 2000 Establishing a Framework for Community Action in the Field of Water Policy.

[13] Ministerio de Agricultura, Alimentación y Medio Ambiente (2015). Royal Decree 817/2015, of 11 September, Which Establishes the Criteria for Monitoring and Assessment of Surface Water Status and Environmental Quality Standards.

[14] European Food Safety Authority (EFSA) (2011). Conclusion on the peer review of the pesticide risk assessment of the active substance terbuthylazine. EFSA J..

[15] Brühl C.A., Bakanov N., Köthe S., Eichler L., Sorg M., Hörren T., Mühlethaler R., Meinel G., Lehmann G.U.C. (2021). Direct pesticide exposure of insects in nature conservation areas in Germany. Sci. Rep..

[16] Mladinic M., Zeljezic D., Shaposhnikov S.A., Collins A.R. (2012). The use of FISH-comet to detect c-Myc and TP 53 damage in extended-term lymphocyte cultures treated with terbuthylazine and carbofuran. Toxicol. Lett..

[17] EU (2019). Commission Implementing Regulation 2019/291 of 19 February 2019 amending Implementing Regulation (EU) No 540/2011 as regards the extension of the approval periods of the active substances 1-naphthylacetamide, 1-naphthylacetic acid, acrinathrin, azoxystrobin, fluazifop p, fluroxypyr, imazalil, kresoxim-methyl, oxyfluorfen, prochloraz, prohexadione, spiroxamine, tefluthrin and terbuthylazine. Off. J. Eur. Union.

[18] Tasca A.L., Puccini M., Fletcher A. (2018). Terbuthylazine and desethylterbuthylazine: Recent occurrence, mobility and removal techniques. Chemosphere.

[19] De la Cruz N., Esquius L., Grandjean D., Magnet A., Tungler A., de Alencastro L.F., Pulgarín C. (2013). Degradation of emergent contaminants by UV, UV/H2O2 and neutral photo-Fenton at pilot scale in a domestic wastewater treatment plant. Water Res..

[20] Xu J., Guo Y., Yang Q., Bai X., Lu R., Liu M., Kuang Z., Zhang L., Li J. (2023). Enhanced cyanogen chloride formation after UV/PS and UV/H2O2 pre-oxidation and chlorination in natural river water. J. Environ. Sci..

[21] Zhang A., Ding Y., Jia A., Park M., Daniels K.D., Nie X., Wu S. (2022). Removal of 26 corticosteroids, potential COVID-19 remedies, at environmentally relevant concentrations in water using UV/free chlorine, UV/monochloramine, and UV/hydrogen peroxide. Environ. Sci. Water Res. Technol..

[22] Álvarez P.M., Quiñones D.H., Terrones I., Rey A., Beltrán F.J. (2016). Insights into the removal of terbuthylazine from aqueous solution by several treatment methods. Water Res..

[23] Lányi K., Dinya Z. (2003). Photodegradation study of some triazine-type herbicides. Microchem. J..

[24] Minero C., Pramauro E., Pelizzetti E., Dolci M., Marchesini A. (1992). Photosensitized transformations of atrazine under simulated sunlight in aqueous humic acid solution. Chemosphere.

[25] Beltran F.J., García-Araya J.F., Acedo B. (1994). Advanced oxidation of atrazine in water—I. Ozonation. Water Res..

[26] Cerreta G., Roccamante M.A., Oller I., Malato S., Rizzo L. (2019). Contaminants of emerging concern removal from real wastewater by UV/free chlorine process: A comparison with solar/free chlorine and UV/H2O2 at pilot scale. Chemosphere.

[27] Sanlaville Y., Guittonneau S., Mansour M., Feicht E.A., Meallier P., Kettrup A. (1996). Photosensitized degradation of terbuthylazine in water. Chemosphere.

[28] Moradi M., Elahinia A., Vasseghian Y., Dragoi E.N., Omidi F., Khaneghah A.M. (2020). A review on pollutants removal by Sono-photo-Fenton processes. J. Environ. Chem. Eng..

[29] Verma M., Haritash A.K. (2019). Degradation of amoxicillin by Fenton and Fenton-integrated hybrid oxidation processes. J. Environ. Chem. Eng..

[30] Legrini O., Oliveros E., Braun A.M. (1993). Photochemical processes for water treatment. Chem. Rev..

[31] Cuerda-Correa E.M., Alexandre M.F., Fernández-González C. (2020). Advanced oxidation processes for the removal of antibiotics from water. An overview. Water.

[32] Prada-Vásquez M.A., Estrada-Flórez S.E., Serna-Galvis E.F., Torres-Palma R.A. (2021). Developments in the intensification of photo-Fenton and ozonation-based processes for the removal of contaminants of emerging concern in Ibero-American countries. Sci. Total Environ..

[33] Feng Y., Qing W., Kong L., Li H., Wu D., Fan Y., Lee P.H., Shih K. (2019). Factors and mechanisms that influence the reactivity of trivalent copper: A novel oxidant for selective degradation of antibiotics. Water Res..

[34] Gallard H., De Laat J., Legube B. (1999). Spectrophotometric study of the formation of iron(III)-hydroperoxy complexes in homogeneous aqueous solutions. Water Res..

[35] Arts A., Schmuhl R., de Groot M.T., van der Schaaf J. (2021). Fast initial oxidation of formic acid by the Fenton reaction under industrial conditions. J. Water Process. Eng..

[36] Haber F., Weiss J. (1934). The catalytic decom position of hydrogen peroxide by Iron salts. Proc. R. Soc. A.

[37] Gultekin I., Ince H.I. (2004). Degradation of Reactive Azo Dyes by UV/H_2_O_2_: Impact of Radical Scavengers. J. Environ. Sci. Health.

[38] Krupińska I. (2020). Impact of the oxidant type on the efficiency of the oxidation and removal of iron compounds from groundwater containing humic substances. Molecules.

[39] Leresche F., Torres-Ruiz J.A., Kurtz T., von Gunten U., Rosario-Ortiz F.L. (2021). Optical properties and photochemical production of hydroxyl radical and singlet oxygen after ozonation of dissolved organic matter. Environ. Sci. Water Resour. Technol..

[40] Liao C.H., Gurol M.D. (1995). Chemical oxidation by photolytic decomposition of hydrogen peroxide. Environ. Sci. Technol..

[41] Guo H.G., Gao N.Y., Chu W.H., Li L., Zhang Y.J., Gu J.S., Gu Y.L. (2013). Photochemical degradation of ciprofloxacin in UV and UV/H_2_O_2_ process: Kinetics, parameters, and products. Environ. Sci. Pollut. Res..

[42] Bottoni P., Grenni P., Lucentini L., Caracciolo A.B. (2013). Terbuthylazine and other triazines in Italian water resources. Microchem. J..

[43] Carra I., Sánches Pérez J.A., Malato S., Autín O., Jefferson B., Jarvis P. (2014). Performance of different advanced oxidation processes for tertiary wastewater treatment to remove the pesticide acetamiprid. J. Chem. Technol. Biotecehnol..

[44] Devi L.G., Munikrishnappa C., Nagaraj B., Rajashekhar E. (2013). Effect of chloride and sulfate ions on the advanced photo Fenton and modified photo Fenton degradation process of Alizarin Red, S. J. Mol. Catal. A Chem..

[45] Templeton M.R., Andrews R.C., Hofmann R. (2006). Impact of iron particles in groundwater on the UV inactivation of bacteriophages MS2 and T4. J. Appl. Microbiol..

[46] Paterlini W.C., Nogueira R.P. (2005). Multivariate analysis of photo-Fenton degradation of the herbicides tebuthiuron, diuron and 2,4-D. Chemosphere.

[47] Canonica S. (2007). Oxidation of aquatic organic contaminants induced by excited triplet states. CHIMIA Int. J. Chem..

[48] Gao H., Zepp R.G. (1998). Factors influencing photoreactions of dissolved organic matter in a coastal river of the southeastern United States. Environ. Sci. Technol..

[49] National Center for Biotechnology Information PubChem Compound Summary for CID 22206, Terbuthylazine; PubChem. https://pubchem.ncbi.nlm.nih.gov/compound/Terbuthylazine.

[50] Acero J.L., Stemmler K., von Gunten U. (2000). Degradation kinetics of atrazine and its degradation products with ozone and OH radicals:  A predictive tool for drinking water treatment. Environ. Sci. Technol..

[51] Sorlini S., Gialdini F., Stefan M. (2014). UV/H_2_O_2_ oxidation of arsenic and terbuthylazine in drinking water. Environ. Monit. Assess..

