# Supplementary material for: Comparative Effect of UV, UV/H2O2 and UV/H2O2/Fe on Terbuthylazine Degradation in Natural and Ultrapure Water
Source: Molecules. 2022 Jul 14;27(14):4507. doi: 10.3390/molecules27144507 (PMC9321380; doi:10.3390/molecules27144507)
Supplement: Supplementary file 1 [file molecules-27-04507-s001.zip › molecules-1747790-supplementary.pdf]

molecules

MDPI

Supplementary Materials

Comparative Effect of UV, UV/H_2O_2 and $UV/H_2O_2/Fe$ on Terbuthylazine Degradation in Natural and Ultrapure Water

José Antonio Andrades, Manuel Lojo-López, Agata Egea-Corbacho * and José María Quiroga

Department of Environmental Technologies, Faculty of Marine and Environmental Sciences, University of Cadiz, Puerto Real, 11510 Cadiz, Spain; antonio.andrade@uca.es (J.A.A.); manuel.lojo@uca.es (M.L.-L.); josemaria.quiroga@uca.es (J.M.Q.)

* Correspondence: agata.egea@uca.es; Tel.: +34-956-016-000

(a)

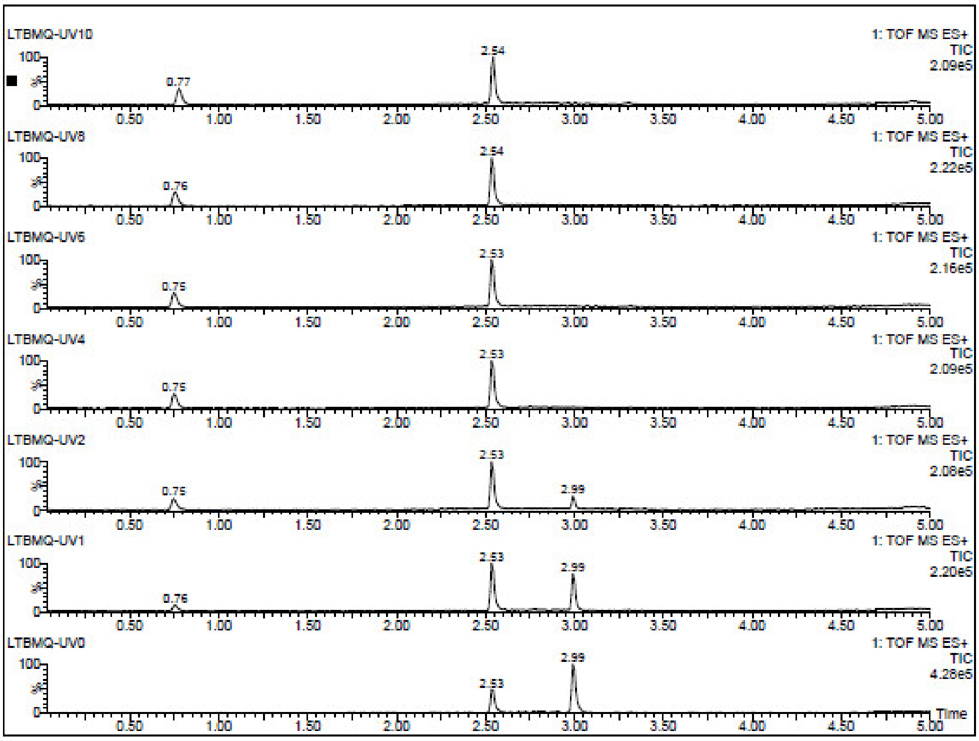

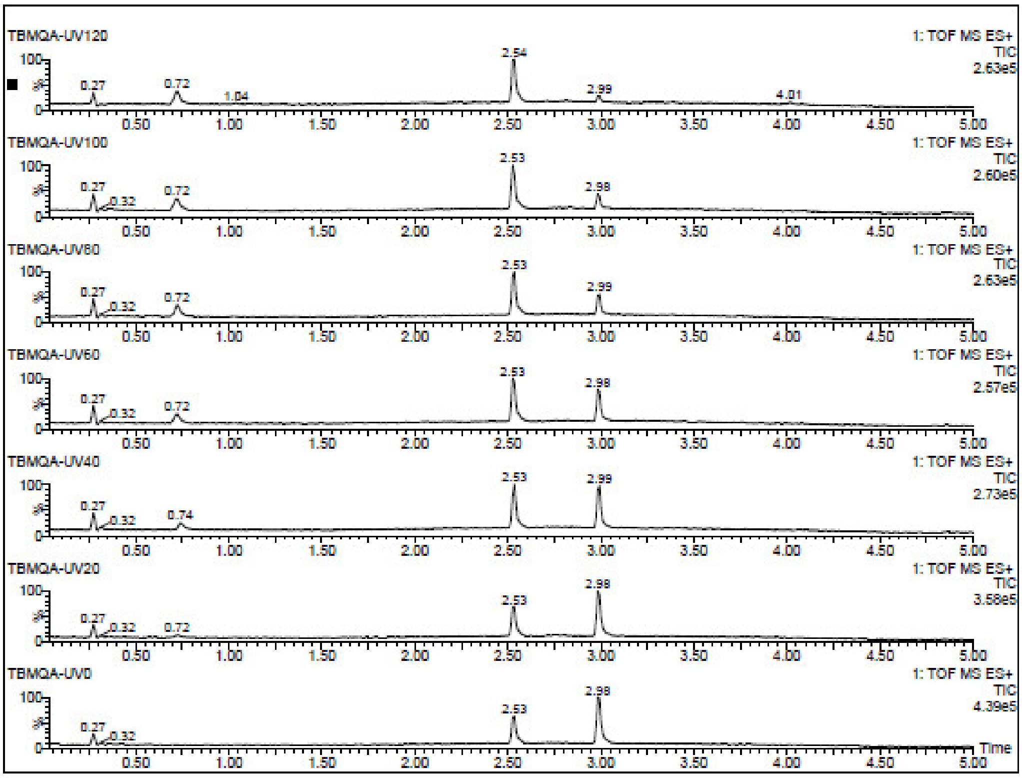

(b)

Figure S1. Chromatogram of terbuthylazine degradation over time in the LUZCHEM reactor (a) and in the semi-continuous reactor (b) after application of UV radiation in ultrapure water.
